# Supplementary material for: Antiretroviral treatment does not prevent extrapulmonary tuberculosis during SIV/Mtb coinfection in macaques
Source: JCI Insight. 2026 Feb 23;11(4):e199314. doi: 10.1172/jci.insight.199314 (PMC12956018; doi:10.1172/jci.insight.199314)
Supplement: Supplemental data [file jciinsight-11-199314-s037.pdf]

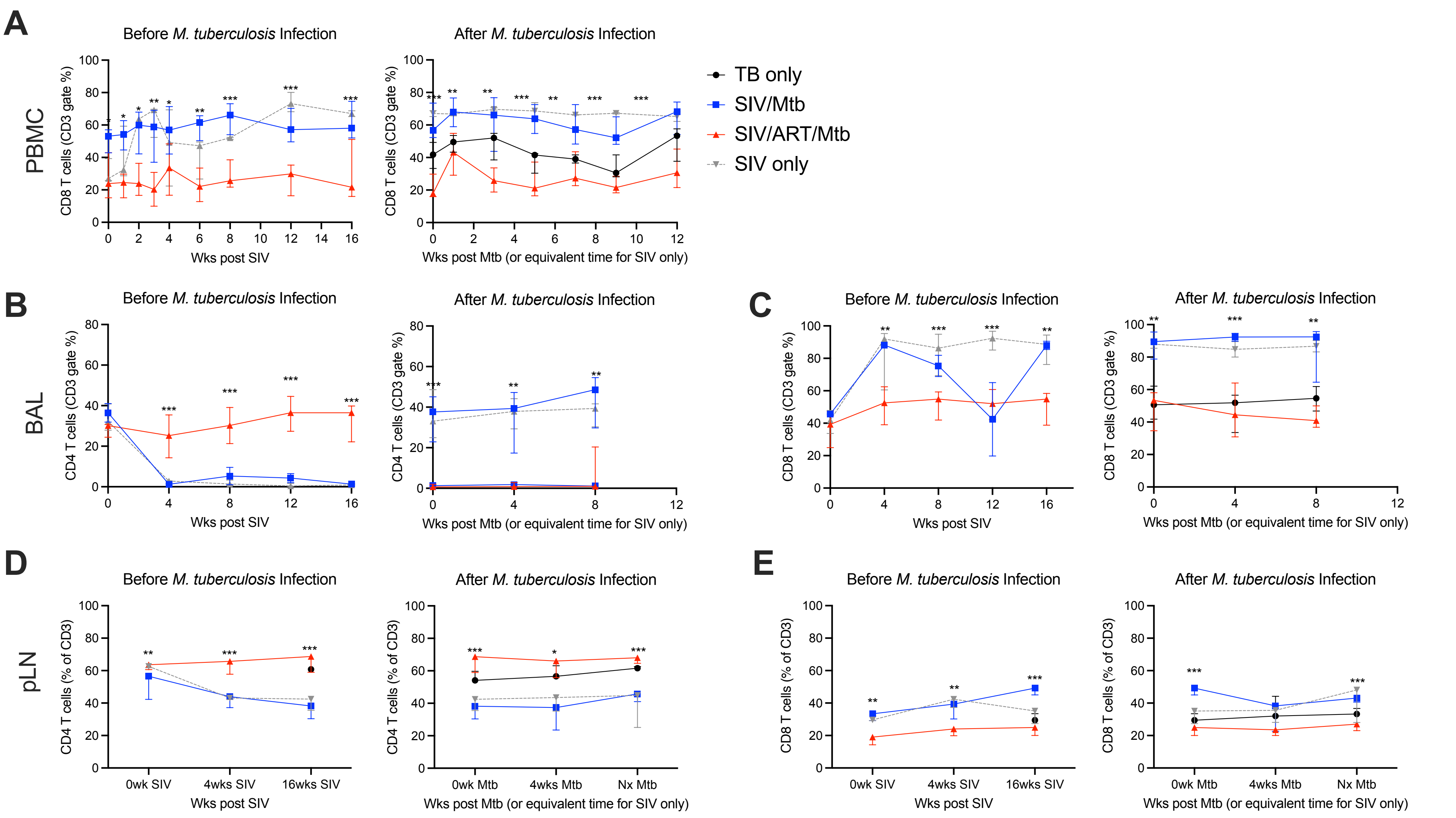

Supplemental Figure 1. A) Serial frequencies of CD8 T cells in the peripheral blood before and after Mtb challenge. B and C) Serial CD4 T cells and CD8 T cells in the airway, D and E) Serial frequencies of CD4 and CD8 T cells in peripheral lymph nodes (pLN). (TB only =9, SIV/TB =9, SIV/ART/TB= 10, SIV only = 3) Median with IQR error bars shown. Statistical analysis was restricted to compare only SIV/ART/TB and SIV/TB groups. Mann-Whitney test run at each time point and adjusted for multiple comparisons by Holm-Šídák method (+: $0.05 < p < 0.10$, *: $0.01 < p < 0.05$, **: $0.001 < p < 0.01$, ***: $p < 0.001$). Black: TB only; Blue: SIV/TB; Red: SIV/ART/TB; Gray: SIV-only.

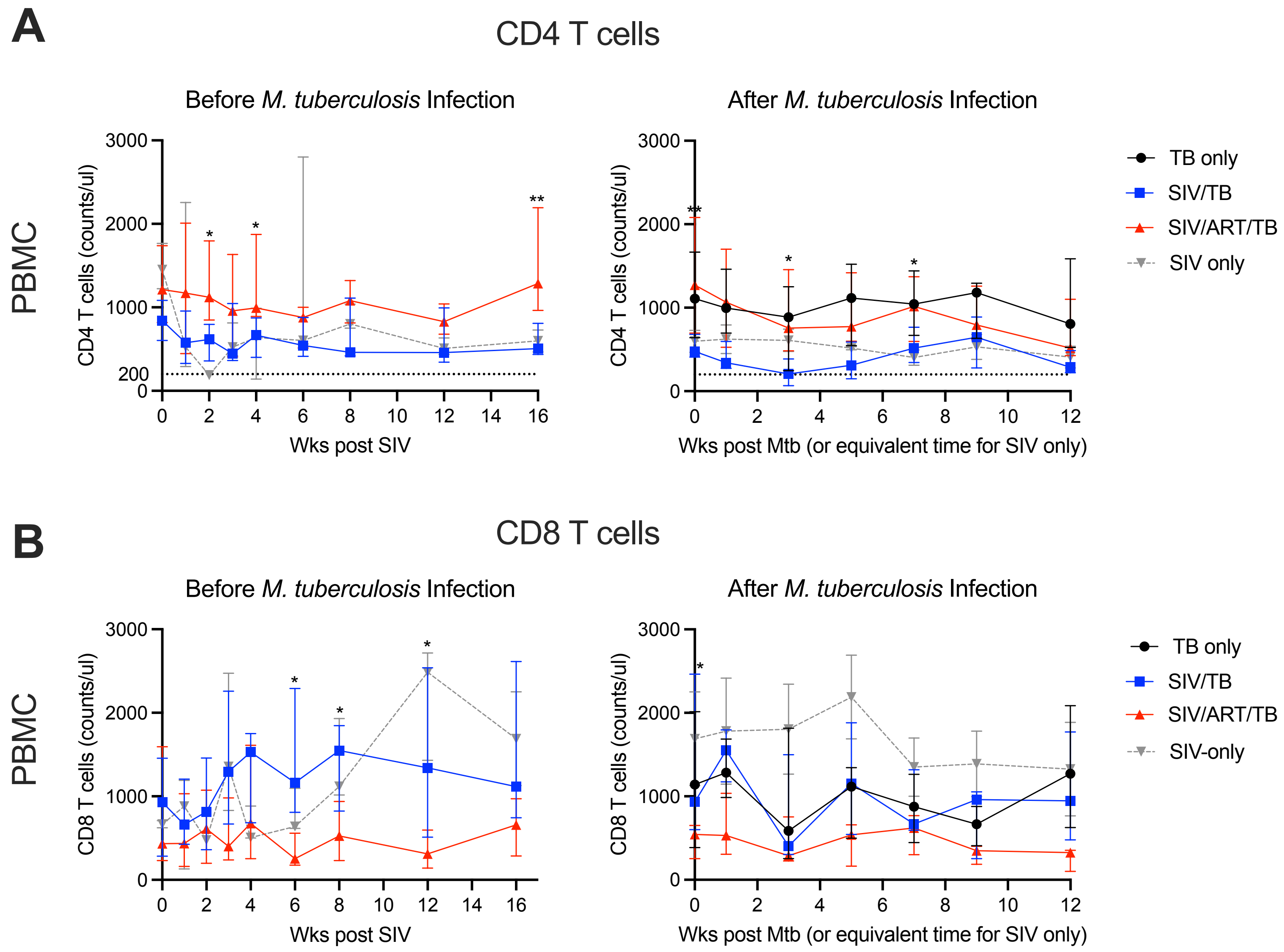

Supplemental Figure 2. Absolute numbers of CD4 (A) and CD8 (B) T cell counts in peripheral blood are shown across groups of animals. (TB only = 9, SIV/TB =9, SIV/ART/TB=10, SIV only = 3). Median with IQR error bars shown. Statistical analysis was restricted to compare only SIV/ART/TB and SIV/TB groups. Mann-Whitney test run at each time point and adjusted for multiple comparisons by Holm-Šídák method (+: $0.05 < p < 0.10$, *: $0.01 < p < 0.05$, **: $0.001 < p < 0.01$, ***: $p < 0.001$). Black: TB only; Blue: SIV/TB; Red: SIV/ART/TB; Gray: SIV-only.

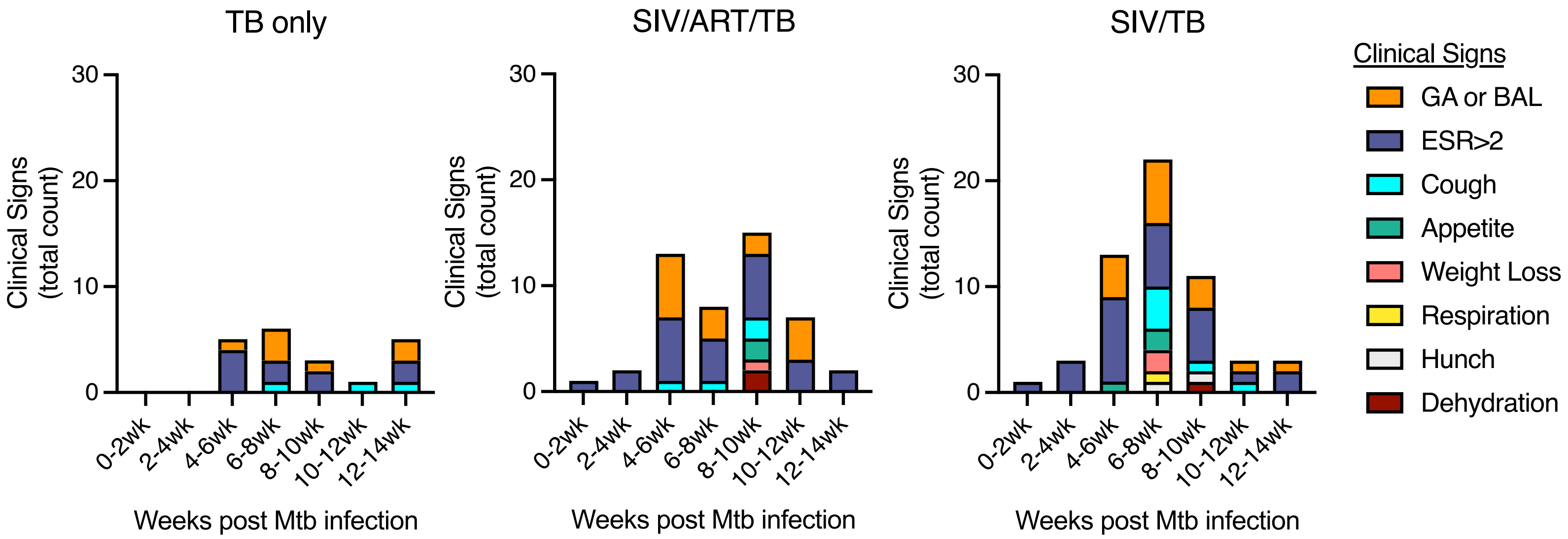

Supplemental Figure 3. Detailed clinical signs for each experimental group is shown by stack graph across each time point. Each stack graph represents the sum of clinical signs within each experimental group at that time point. TB only (n=9), SIV/ART/TB (n=10), SIV/TB (n=9).

A

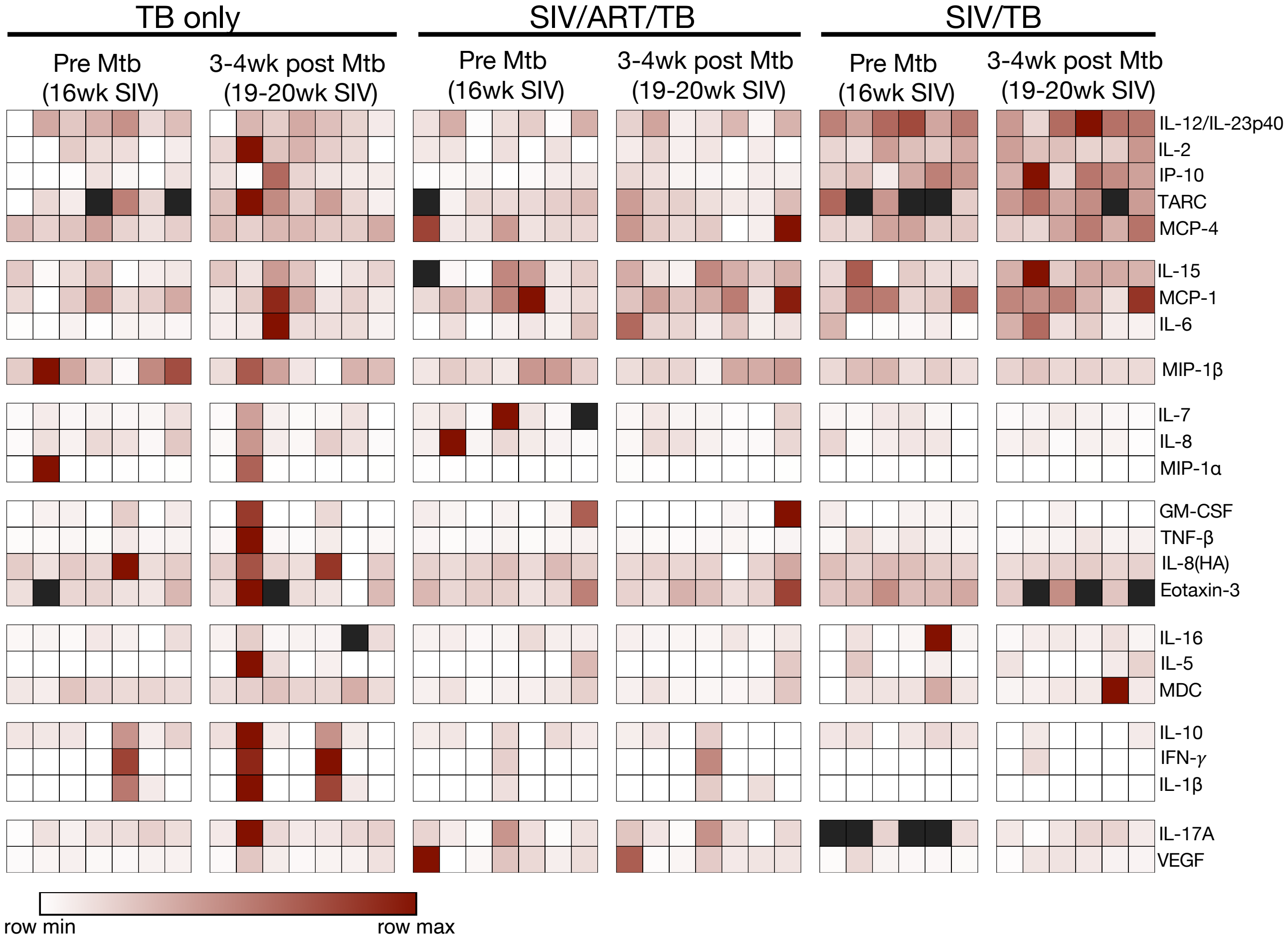

B

	Pre Mtb		3-4wk post Mtb	
	p-value	95% CI of the mean difference	p-value	95% CI of the mean difference
**IL-12/IL-23p40**				
SIV/ART/TB vs SIV/TB	0.0008	42.82 to 173.1	0.0012	38.99 to 169.3
TB only vs SIV/TB	0.012	15.92 to 146.2	0.001	40.28 to 170.5
TB only vs SIV/ART/TB	0.549	-89.47 to 35.68	0.9986	-61.29 to 63.86
**IL-2**				
SIV/ART/TB vs SIV/TB	0.0826	-0.020 to 0.41	0.0402	0.008 to 0.44
TB only vs SIV/TB	0.1159	-0.035 to 0.398	0.998	-0.22 to 0.21
TB only vs SIV/ART/TB	0.9834	-0.223 to 0.193	0.0273	-0.43 to -0.02
**IP-10**				
SIV/ART/TB vs SIV/TB	0.0106	149.6 to 1285	0.0001	534.0 to 1669
TB only vs SIV/TB	0.0106	150.1 to 1285	0.0026	274.5 to 1410
TB only vs SIV/ART/TB	>0.9999	-544.9 to 545.9	0.4811	-805.0 to 285.8
**TARC**				
SIV/ART/TB vs SIV/TB	0.1495	-0.2785 to 2.238	0.0735	-0.077 to 2.007
TB only vs SIV/TB	0.2029	-0.3783 to 2.221	0.7243	-0.7185to 1.366
TB only vs SIV/ART/TB	0.9901	-1.136 to 1.019	0.2342	-1.593 to 0.309
**MCP-4**				
SIV/ART/TB vs SIV/TB	0.9982	-8.377 to 7.994	0.8034	-6.073 to 10.30
TB only vs SIV/TB	0.9561	-7.232 to 9.140	0.5891	-4.884 to 11.49
TB only vs SIV/ART/TB	0.9323	-6.719 to 9.011	0.9273	-6.676 to 9.054

Supplemental Figure 4. Cytokine and chemokine expression within plasma. (A) Each column represents a macaque and the column groups are divided into their specific infection cohort (TB-only, SIV/ART/TB, and SIV/TB) and time point. Each row represents a different cytokine or chemokine, ranging from smallest (white) to greatest (dark red). The cytokines were grouped using hierarchical clustering on one-minus-Kendall's correlation (and average linkage method). Black squares represent missing data points. This figure was created using Morpheus, https://software.broadinstitute.org/morpheus/. (B) Table showing results of pairwise comparisons of the top cluster (top 5 rows). Tukey's adjusted p-values shown with 95 % CI (confidence intervals) of the mean difference between treatment groups.

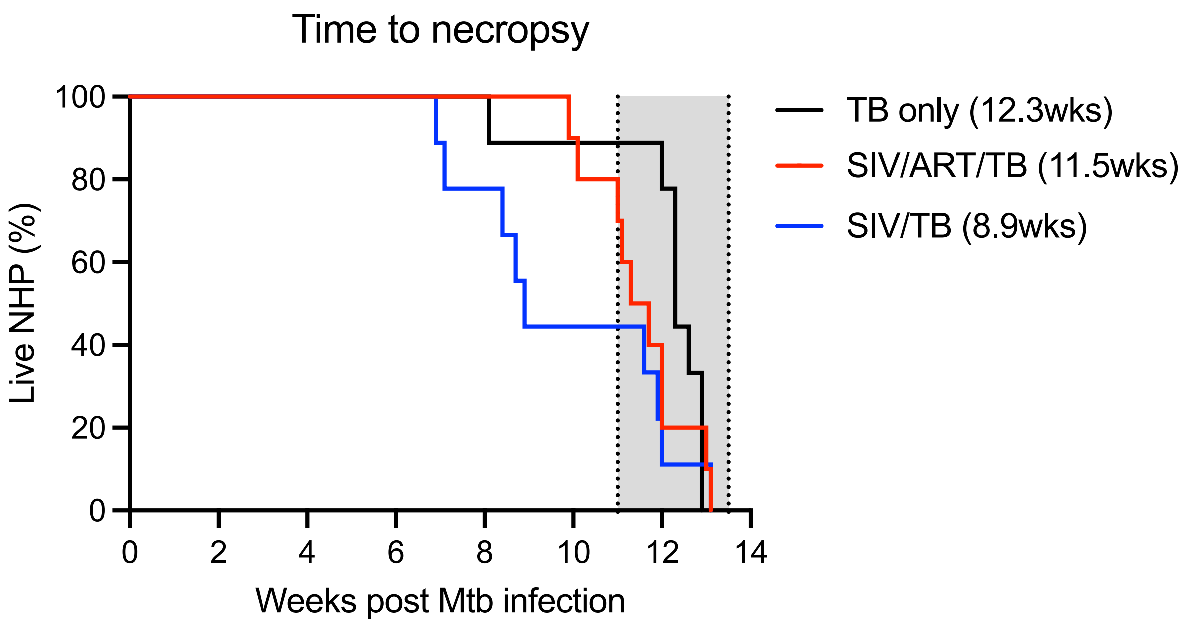

Supplemental Figure 5. Proportion of animals surviving to declared endpoint (12 wk) after Mtb challenge. Median weeks of Mtb infection are shown in ( ). Grayed area indicates time of planned necropsy.

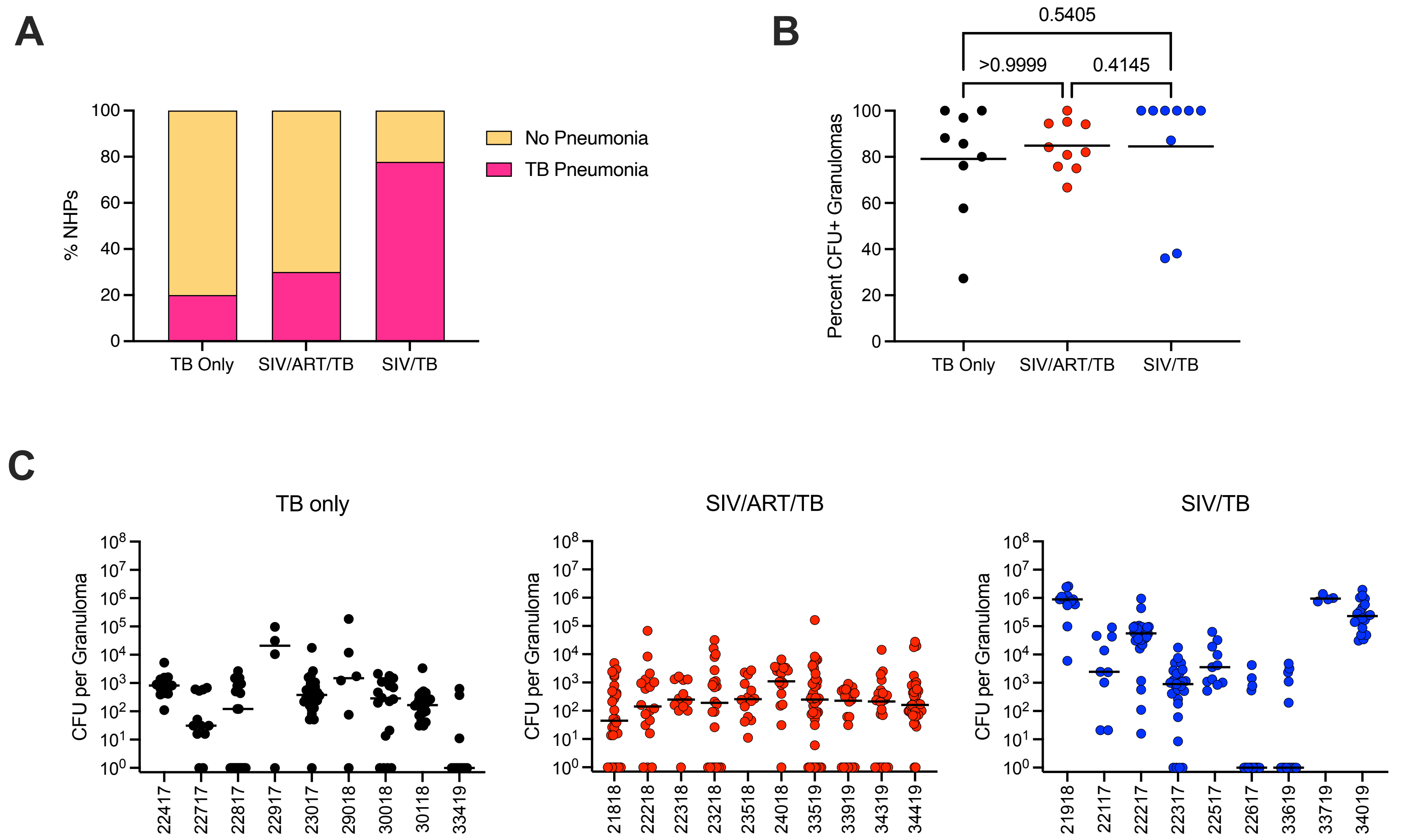

Supplemental Figure 6. A) Proportion of NHPs in each experimental group with TB pneumonia determined by PET CT, Fisher's Exact test p-value 0.0278. B) Percentage of granulomas and clusters with viable Mtb growth. Each dot represents an animal; lines represent means. Kruskal-Wallis test with Dunn's multiple comparison adjusted p-values reported. C) Individual bacterial burden (CFU) per granuloma by animal and group. Each dot represents a granuloma; lines represent median per animal.

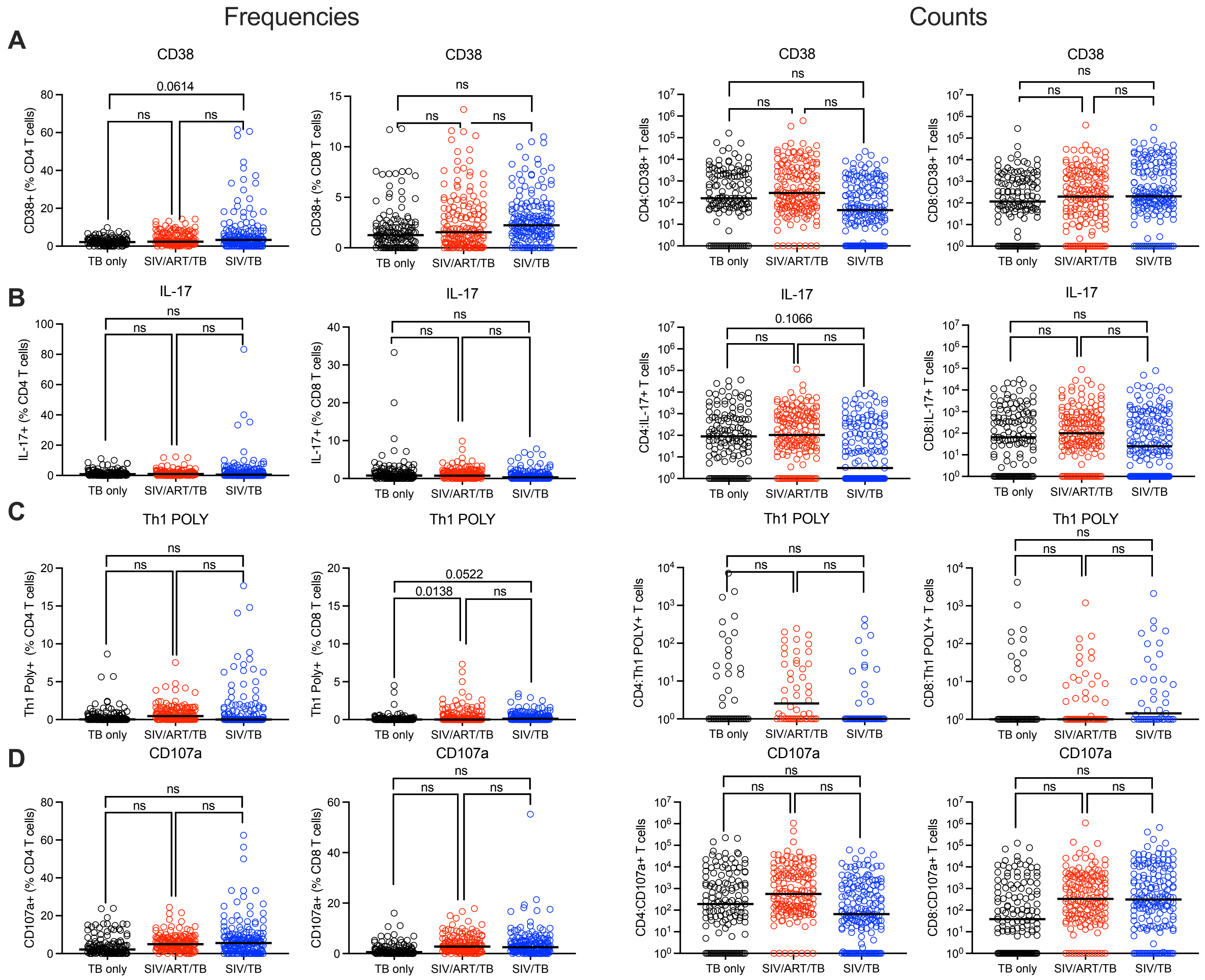

Supplemental Figure 7. Immunophenotyping of granuloma specific CD8 and CD4 T cells based on frequencies (left) and absolute cell numbers (right columns). Th1 POLY represents cells that express two or more of the following: IFN-g, TNF, IL-2. Each circle represents a granuloma; lines are medians. Mixed effect model (animal as a random effect and treatment group as a fixed effect) was used; Tukey HSD adjusted p-values (for $p < 0.10$) reported. "ns" means not significant ($p > 0.10$).

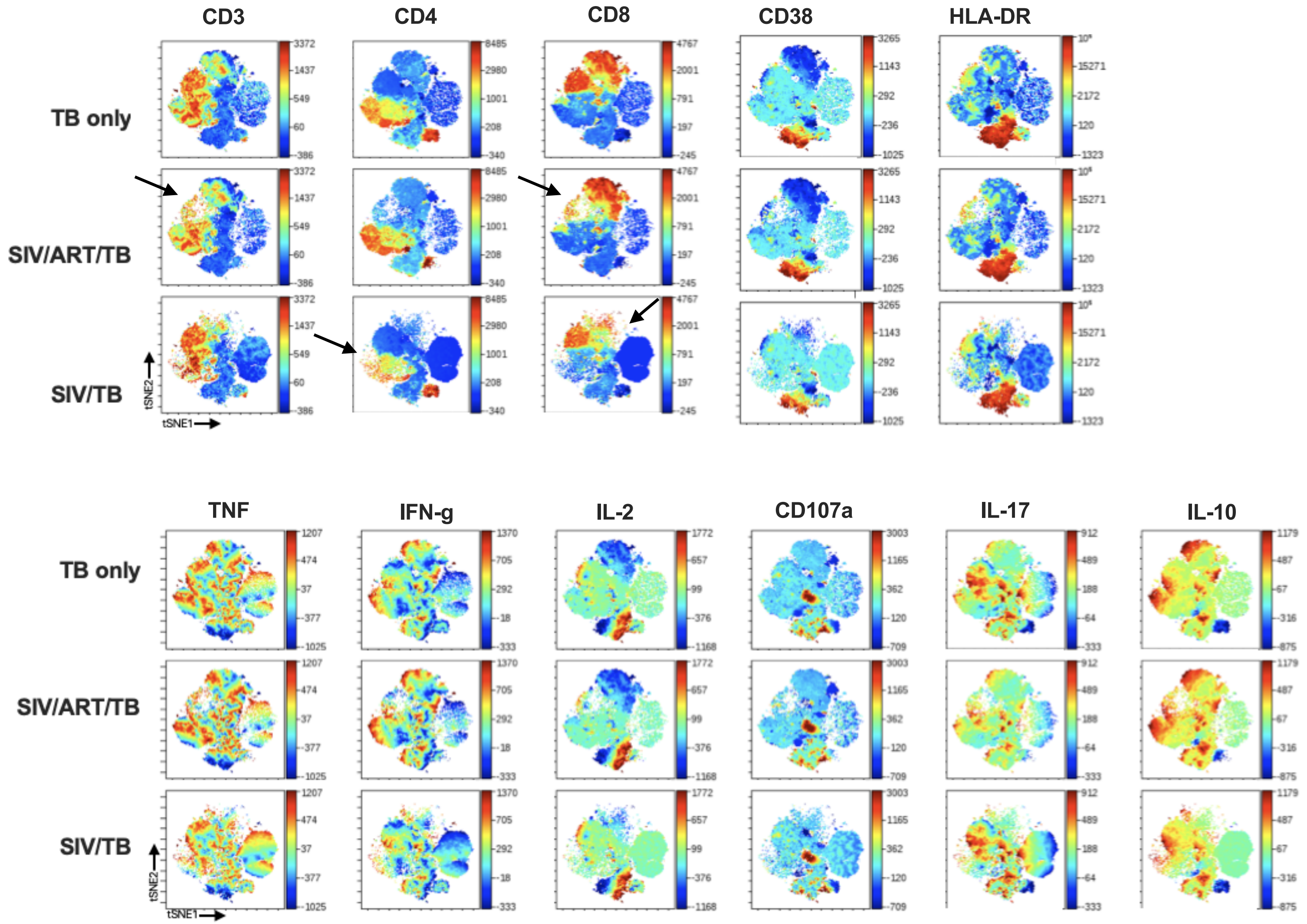

Supplemental Figure 8. t-SNE visualization of cellular markers among granuloma-specific lymphocytes. Pseudocolored maps show the relative expression of respective markers where red represents high expression and blue is low expression. Arrows highlight T cell populations based on phenotypic characteristics that differ between experimental groups. (n=39, 43, and 19 lung granulomas from 3 SIV/TB, 3 SIV/ART/TB, and 2 TB-only NHPs, respectively).

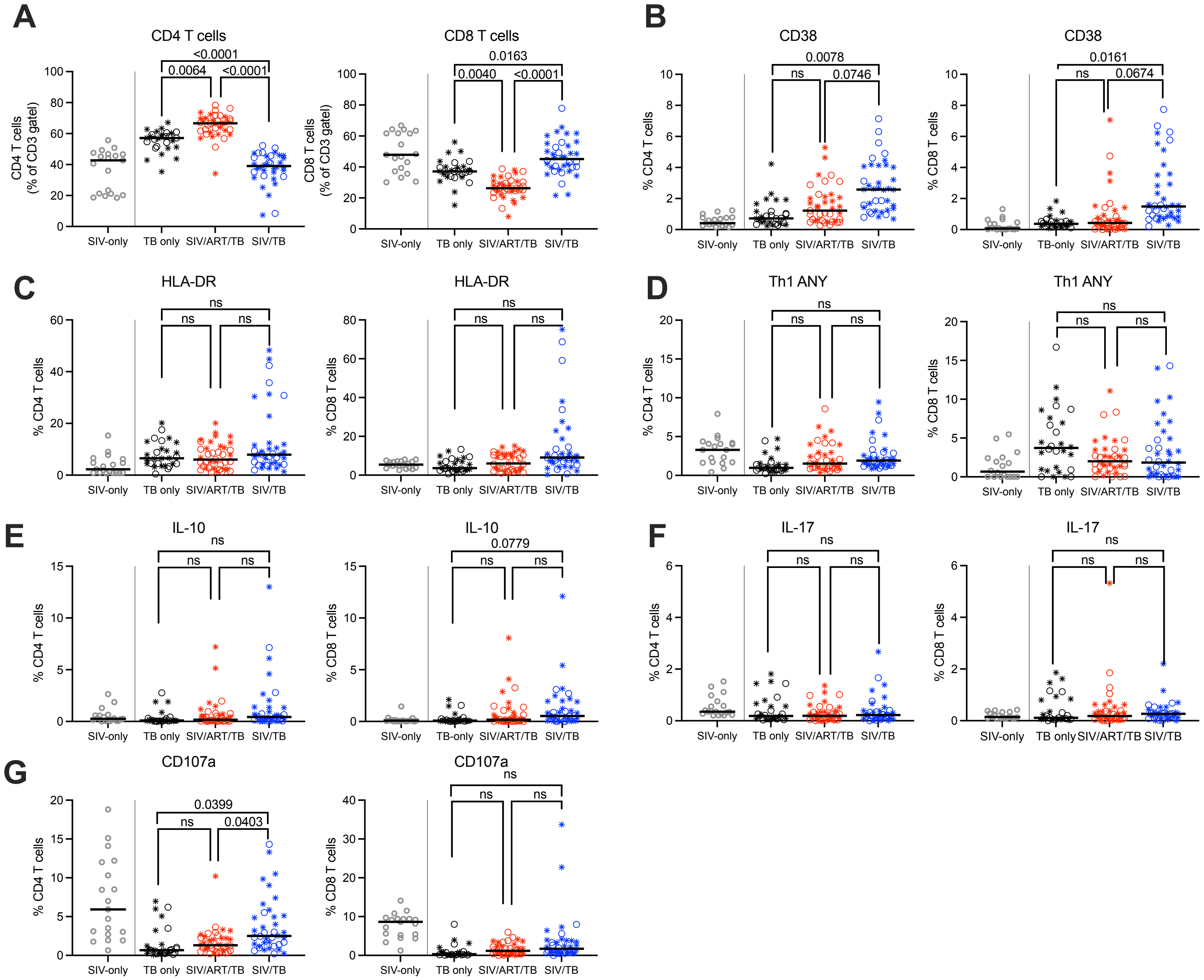

Supplemental Figure 9. Frequency and functional characteristics of CD4 and CD8 T cells from thoracic lymph nodes (LN). Th1 (any) represents at least one of the following: IFN-g, TNF, or IL-2. Each circle is a mediastinal LN. Stars represent LN with granuloma. Lines are medians. Mixed effect model (animal as a random effect and treatment group as a fixed effect) was used; Tukey HSD adjusted p-values (for $p < 0.10$) reported. “ns” means not significant ($p > 0.10$). SIV only groups is shown for visual representation without statistical comparison. Statistical comparison is performed across TB only, SIV/ART/TB and SIV/TB groups.

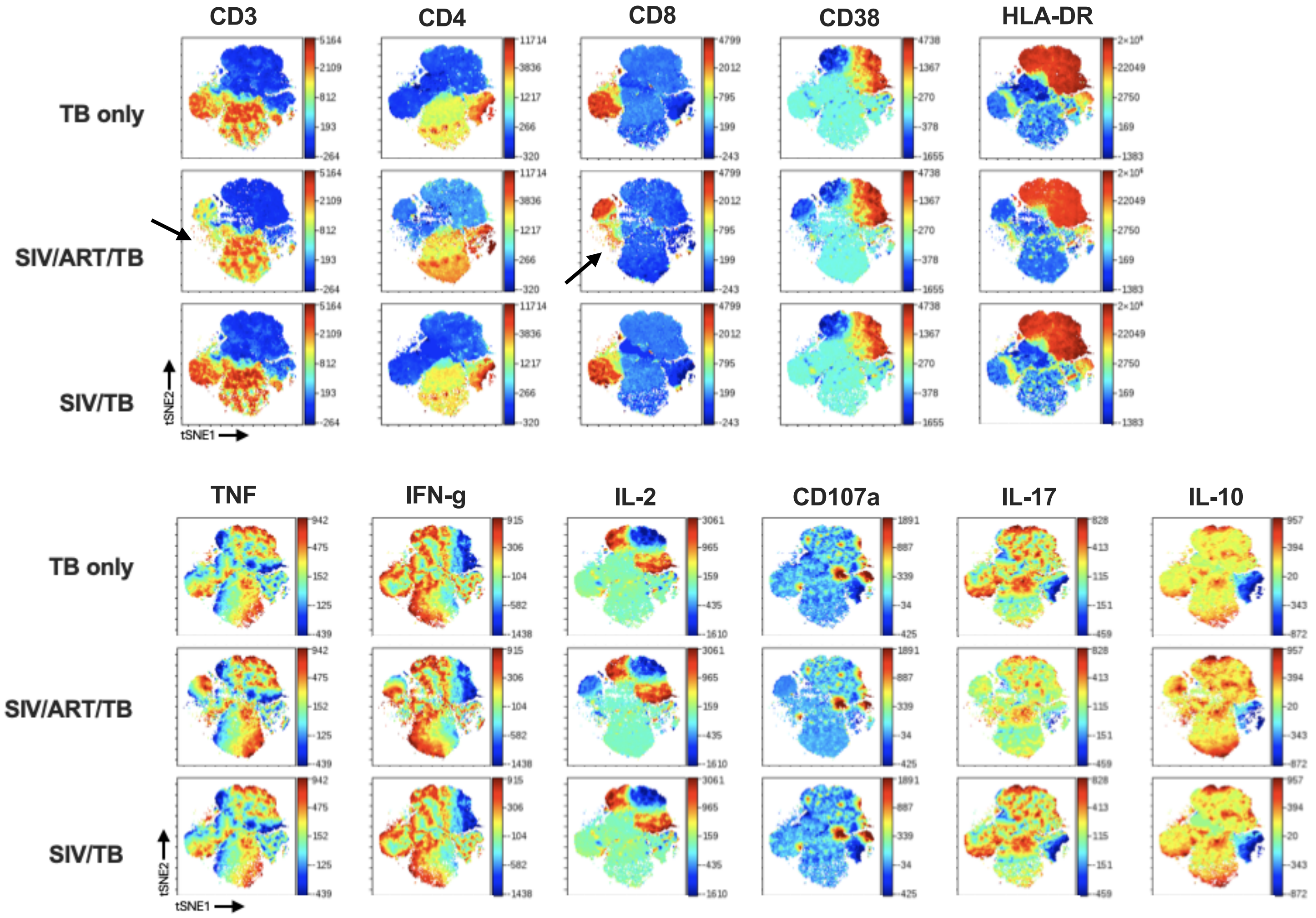

Supplemental Figure 10. t-SNE visualization of cellular markers lymphocytes in thoracic LNs. Pseudocolored maps show the relative expression of respective markers where red represents high expression and blue is low expression. Arrows highlight T cell populations based on phenotypic characteristics that differ between experimental groups. (n=12, 14, and 4 thoracic LN from 3 SIV/TB, 3 SIV/ART/TB, and 2 TB-only NHPs, respectively).

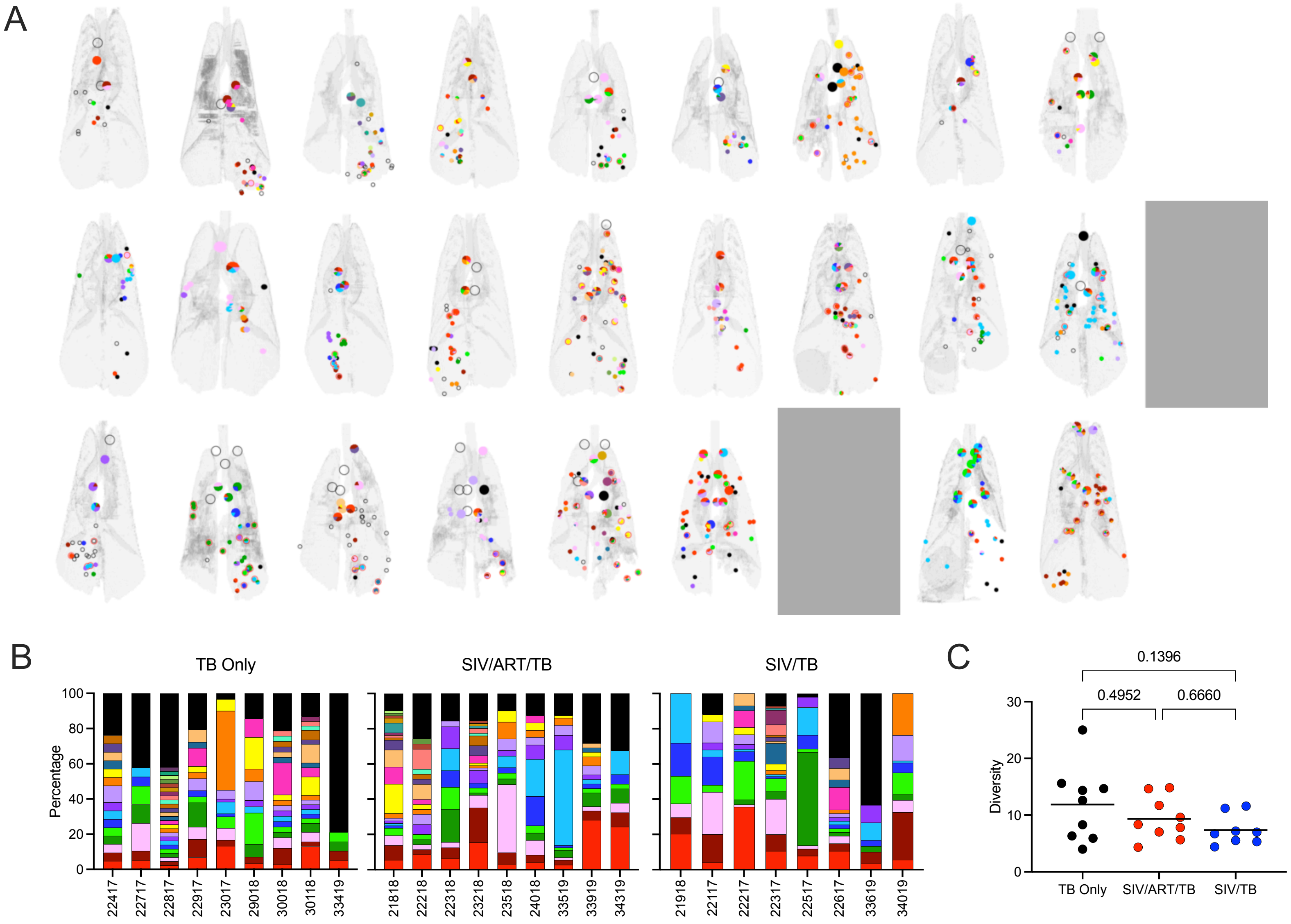

Supplemental Figure 11: A) Three-dimensional renderings of lungs with location of barcodes. Small dots represent granulomas; large circles represent thoracic lymph nodes; open circles represent sterile tissue. Two animals did not have viable barcodes; these are represented by gray boxes. B) Distribution of barcodes in the lungs each animal. Each color represents a barcode; black bars represent sterile samples. C) Diversity of barcodes (measured by the effective number quantified using the Shannon entropy index) in the lungs. Each dot represents an animal and lines represent group means. Groups were compared using ordinary one-way ANOVA with Tukey's multiple comparison adjusted p-values shown. TB only (n=9), SIV/ART/TB (n=9), SIV/TB (n=8).

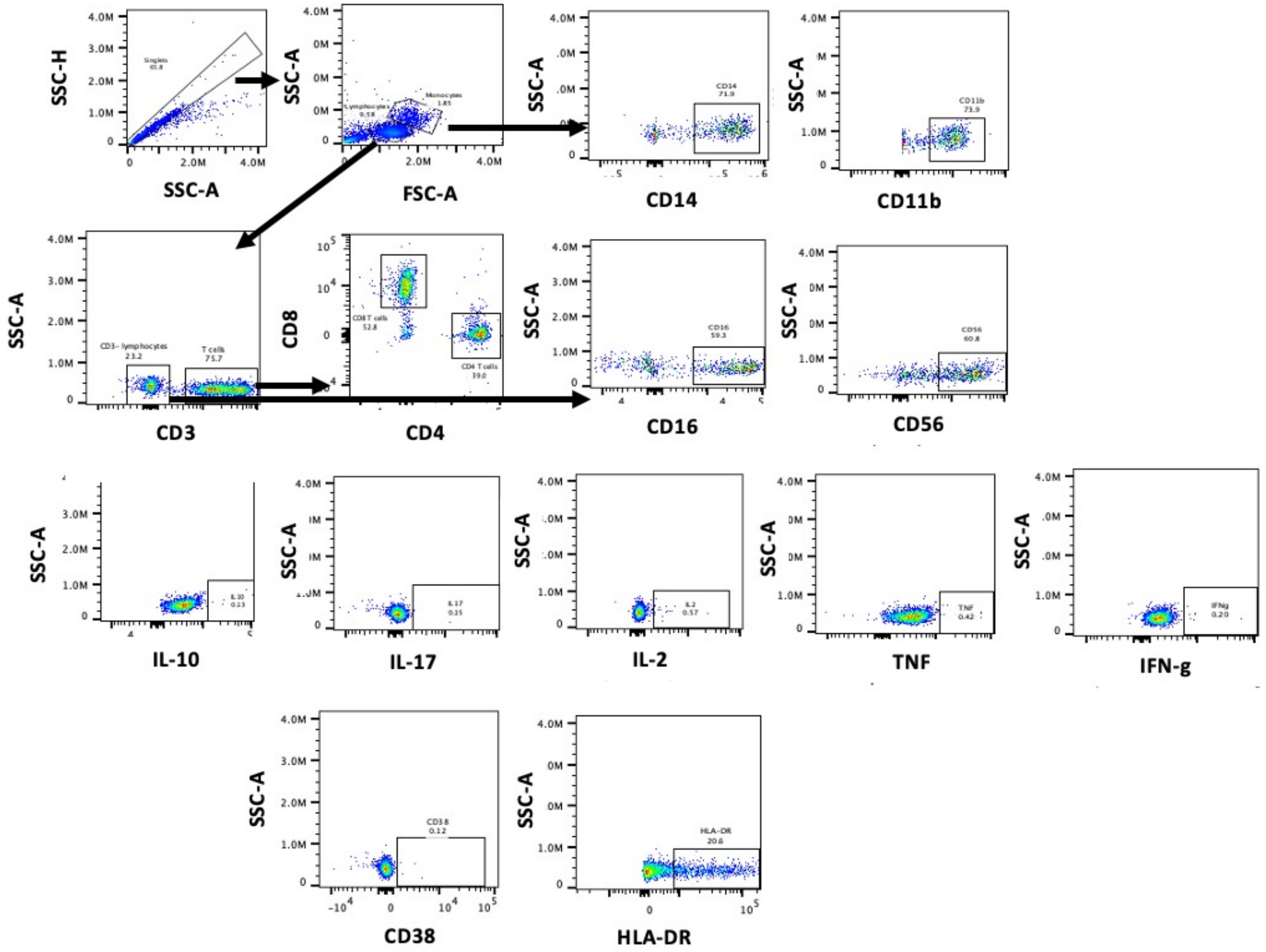

Supplemental Figure 12. Spectral flow gating strategy for PBMC and lymph node (CD107A) stimulated with ESAT6 and CFP10.

Animal ID	Cohort	Gender	Age (yrs)	Weeks infected with SIV	Necropsy (wks post Mtb)
22917	TB-only	F	8	NA	8
29018	TB-only	M	5	NA	12
30018	TB-only	M	5	NA	12
30118	TB-only	M	5	NA	12
33419	TB-only	M	6	NA	12
23017	TB-only	F	6	NA	13
22417	TB-only	M	6	NA	13
22717	TB-only	M	7	NA	13
22817	TB-only	M	7	NA	13
34419	SIV/ART/TB	M	7	26	10
34319	SIV/ART/TB	M	6	26	10
23218	SIV/ART/TB	M	8	27	11
33519	SIV/ART/TB	F	5	27	11
24018	SIV/ART/TB	M	9	27	11
23518	SIV/ART/TB	M	6	28	12
21818	SIV/ART/TB	M	5	28	12
22218	SIV/ART/TB	M	7	28	12
22318	SIV/ART/TB	M	6	29	13
33919	SIV/ART/TB	M	5	29	13
22217	SIV/TB	M	7	23	7
21918	SIV/TB	M	5	23	7
34019	SIV/TB	M	6	24	8
33719	SIV/TB	M	6	25	9
22317	SIV/TB	M	6	25	9
22117	SIV/TB	M	8	28	12
22517	SIV/TB	M	8	28	12
33619	SIV/TB	F	6	28	12
22617	SIV/TB	M	8	29	13
33819	SIV-only	M	6	11	NA
34119	SIV-only	M	6	29	NA
34219	SIV-only	M	6	29	NA

Supplemental Table 1. Animal identification number, experimental group, gender, age (in years), weeks of SIV infection, and time to necropsy (weeks post Mtb infection) are shown.

Marker	Color	Clone	Company	Samples
CD11b	APC-Cy7	ICRF44	BD Bio Sciences	BAL
CD16	PE-Cy5	3G8	BD Pharmingen	BAL
CD206	PerCPCy5.5	19.2	BD Bio Sciences	BAL
CD3	PE-CF594	SP34	BD Pharmingen	BAL
CD4	BV510	L200	BD Horizons	BAL
CD45	APC	D058 1283	BD Bio Sciences	BAL
CD56	PE-Cy5	HCD56	Biolegend	BAL
CD8	Biotin:Streptavidin-AF700	SK1	BD Bio Sciences	BAL
HLA-DR	AF700	G46 G	Fisher Scientific	BAL
IFN-g	FITC	B27	BD Bio Sciences	BAL
IL-10	efluor450	JES34A3	eBiosciences	BAL
TNF	FITC	Mab11	BD Bio Sciences	BAL
CD11b	PECy7	ICRF44	BD Bio Sciences	PBMC
CD14	BV768	M5E2	BD Bio Sciences	PBMC
CD16	BUV563	3G8	Biolegend	PBMC
CD3	APC-Cy7	SP34	BD Pharmingen	PBMC
CD38	PE	HIT2	Biolegend	PBMC
CD4	BV510	L200	BD Horizons	PBMC
CD56	PeCy5	FAB1059B	RD Systems	PBMC
CD8	Biotin:Streptavidin-AF700	SK1	BD Bio Sciences	PBMC
HLA-DR	PE-CF594	G46 G	Fisher Scientific	PBMC
IFN-g	AF700	B27	BD Bio Sciences	PBMC
IL-10	efluor450	JES34A3	eBiosciences	PBMC
IL-17	FITC	eBio64CAP17	eBiosciences	PBMC
IL-2	PerCPCy5.5	MQ1-17H12	BD Bio Sciences	PBMC
TNF	APC	Mab11	BD Bio Sciences	PBMC
CD107a	PE	eBioH43	H4A3	Tissue
CD11b	PeCy7	ICRF44	BD Bio Sciences	Tissue
CD3	APC-Cy7	SP34	BD Pharmingen	Tissue
CD38	PeCy5	HIT2	Biolegend	Tissue
CD4	BV510	L200	BD Horizons	Tissue
CD8	Biotin:Streptavidin-AF700	SK1	BD Bio Sciences	Tissue
HLA-DR	PE-CF594	G46 G	Fisher Scientific	Tissue
IFN-g	AF700	B27	BD Bio Sciences	Tissue
IL-10	efluor450	JES34A3	eBiosciences	Tissue
IL-17	FITC	eBio64CAP17	eBiosciences	Tissue
IL-2	PerCPCy5.5	MQ1-17H12	BD Bio Sciences	Tissue
TNF	APC	Mab11	BD Bio Sciences	Tissue

Supplemental Table 2. Spectral flow markers with respective fluorophore, clone, company and sample type shown.
